# 1-[4-(3-Chloro­prop­oxy)-2-hydroxy­phen­yl]ethanone

**DOI:** 10.1107/S1600536809051411

**Published:** 2009-12-04

**Authors:** Ya-Tuan Ma, Jing-Jing Wang, Xi-Wang Liu, Sheng-Xiang Yang, Jin-Ming Gao

**Affiliations:** a College of Life Sciences, Northwest A&F University, Yangling Shaanxi 712100, People’s Republic of China; b College of Science, Northwest A&F University, Yangling Shaanxi 712100, People’s Republic of China

## Abstract

The title compound, C_11_H_13_ClO_3_, has been obtained in the reaction of 2, 4-dihydroxy­lacetonephenone, potassium carbonate and 1-bromo-3-chloro-hexane. The hydr­oxy group is involved in an intra­molecular O—H⋯O hydrogen bond. The crystal packing exhibits no significantly short inter­molecular contacts

## Related literature

For background to the Williamson reaction in organic synthesis, see: Dermer (1934 ▶). For a related structure, see: Schlemper (1986 ▶).
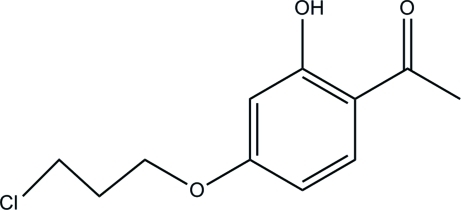


## Experimental

### 

#### Crystal data

C_11_H_13_ClO_3_
*M* *_r_* = 228.66
Orthorhombic, 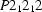

*a* = 18.620 (2) Å
*b* = 11.963 (11) Å
*c* = 5.0240 (6) Å
*V* = 1119.1 (11) Å^3^
*Z* = 4
Mo *K*α radiation
μ = 0.33 mm^−1^
*T* = 298 K
0.49 × 0.44 × 0.43 mm

#### Data collection

Bruker SMART APEX CCD area-detector diffractometer
Absorption correction: multi-scan (*SADABS*; Sheldrick, 1996 ▶) *T*_min_ = 0.857, *T*_max_ = 0.873
4851 measured reflections
1946 independent reflections
1556 reflections with *I* > 2σ(*I*)
*R*_int_ = 0.054

#### Refinement

*R*[*F* ^2^ > 2σ(*F* ^2^)] = 0.042
*wR*(*F* ^2^) = 0.103
*S* = 1.03
1946 reflections
138 parameters
H-atom parameters constrained
Δρ_max_ = 0.22 e Å^−3^
Δρ_min_ = −0.19 e Å^−3^
Absolute structure: Flack (1983 ▶), 761 Friedel pairs
Flack parameter: −0.16 (10)

### 

Data collection: *SMART* (Siemens, 1996 ▶); cell refinement: *SAINT* (Siemens, 1996 ▶); data reduction: *SAINT*; program(s) used to solve structure: *SHELXS97* (Sheldrick, 2008 ▶); program(s) used to refine structure: *SHELXL97* (Sheldrick, 2008 ▶); molecular graphics: *SHELXTL* (Sheldrick, 2008 ▶); software used to prepare material for publication: *SHELXTL*.

## Supplementary Material

Crystal structure: contains datablocks I, global. DOI: 10.1107/S1600536809051411/cv2661sup1.cif

Structure factors: contains datablocks I. DOI: 10.1107/S1600536809051411/cv2661Isup2.hkl

Additional supplementary materials:  crystallographic information; 3D view; checkCIF report

## Figures and Tables

**Table 1:** Hydrogen-bond geometry (Å, °)

*D*—H⋯*A*	*D*—H	H⋯*A*	*D*⋯*A*	*D*—H⋯*A*
O2—H2⋯O1	0.82	1.85	2.570 (3)	146

**Fig. 1.**
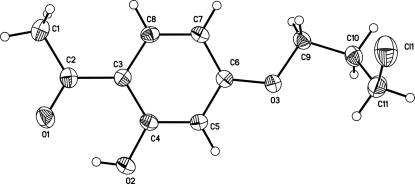
The molecular structure of (I) with atomic numbering and 30% probability displacement ellipsoids.

## supplementary crystallographic information

### Comment

The Williamson reaction is a very useful transformation in organic synthesis since the products are of value in both industrial and academic applications. It usually involves the employment of an alkali-metal salt of the hydroxy compound and an alkylhalide (Dermer, 1934).

In this paper, we present the title compound, (I), which was synthesized by the reaction of 2, 4-dihydroxylacetonephenone, potassium carbonate and 1-bromo-3-chloro-hexane. In (I) (Fig. 1), the bond lengths and angles are normal and comparable to those observed in the related structure (Schlemper, 1986). The dihedral angle between the benzene ring C3-C8 and the plane O3C9C10 is 3.82 (4)°. The crystal packing exhibits no significantly short intermolecular contacts

### Experimental

2, 4-Dihydroxylacetonephenone (3 mmol), potassium carbonate (6 mmol), 1-bromo-3-chloro-hexane (3 mmol), and 10 ml acetone were mixed in 50 ml flask. After 4 h stirring at 373 K, the crude product was obtained. The crystals were obtained by recrystallization from n-hexane/ethyl acetate. Elemental analysis: calculated for C_11_H_13_ClO_3_: C 55.96, H 5.17%; found: C 55.88, H 5.25,%.

### Refinement

All H atoms were positioned geometrically, with O—H= 0.82 Å, C—H=0.93- 0.97 Å, and refined as riding, with *U*_iso_(H)=1.2–1.5*U*_eq_(C, O).

### Crystal data

**Table:**

C_11_H_13_ClO_3_	*D*_x_ = 1.357 Mg m^−^^3^
*M**_r_* = 228.66	Mo *K*α radiation, λ = 0.71073 Å
Orthorhombic, *P*2_1_2_1_2	Cell parameters from 1957 reflections
*a* = 18.620 (2) Å	θ = 2.2–25.7°
*b* = 11.963 (11) Å	µ = 0.33 mm^−^^1^
*c* = 5.0240 (6) Å	*T* = 298 K
*V* = 1119.1 (11) Å^3^	Block, colourless
*Z* = 4	0.49 × 0.44 × 0.43 mm
*F*(000) = 480	

### Data collection

**Table:**

Bruker Smart APEX CCD area-detector diffractometer	1946 independent reflections
Radiation source: fine-focus sealed tube	1556 reflections with *I* > 2σ(*I*)
graphite	*R*_int_ = 0.054
phi and ω scans	θ_max_ = 25.0°, θ_min_ = 2.0°
Absorption correction: multi-scan (*SADABS*; Sheldrick, 1996)	*h* = −18→22
*T*_min_ = 0.857, *T*_max_ = 0.873	*k* = −9→14
4851 measured reflections	*l* = −5→5

### Refinement

**Table:**

Refinement on *F*^2^	Secondary atom site location: difference Fourier map
Least-squares matrix: full	Hydrogen site location: inferred from neighbouring sites
*R*[*F*^2^ > 2σ(*F*^2^)] = 0.042	H-atom parameters constrained
*wR*(*F*^2^) = 0.103	*w* = 1/[σ^2^(*F*_o_^2^) + (0.0468*P*)^2^ + 0.0435*P*] where *P* = (*F*_o_^2^ + 2*F*_c_^2^)/3
*S* = 1.03	(Δ/σ)_max_ < 0.001
1946 reflections	Δρ_max_ = 0.22 e Å^−^^3^
138 parameters	Δρ_min_ = −0.19 e Å^−^^3^
0 restraints	Absolute structure: Flack (1983), 761 Friedel pairs
Primary atom site location: structure-invariant direct methods	Flack parameter: −0.16 (10)

### Special details

**Table:**

Geometry. All e.s.d.'s (except the e.s.d. in the dihedral angle between two l.s. planes) are estimated using the full covariance matrix. The cell e.s.d.'s are taken into account individually in the estimation of e.s.d.'s in distances, angles and torsion angles; correlations between e.s.d.'s in cell parameters are only used when they are defined by crystal symmetry. An approximate (isotropic) treatment of cell e.s.d.'s is used for estimating e.s.d.'s involving l.s. planes.
Refinement. Refinement of *F*^2^ against ALL reflections. The weighted *R*-factor *wR* and goodness of fit *S* are based on *F*^2^, conventional *R*-factors *R* are based on *F*, with *F* set to zero for negative *F*^2^. The threshold expression of *F*^2^ > σ(*F*^2^) is used only for calculating *R*-factors(gt) *etc*. and is not relevant to the choice of reflections for refinement. *R*-factors based on *F*^2^ are statistically about twice as large as those based on *F*, and *R*- factors based on ALL data will be even larger.

### Fractional atomic coordinates and isotropic or equivalent isotropic displacement parameters (Å^2^)

**Table:**

	*x*	*y*	*z*	*U*_iso_*/*U*_eq_	
O3	0.61841 (8)	0.94594 (14)	−0.1602 (4)	0.0460 (5)	
O1	0.81896 (9)	1.15126 (15)	0.7343 (4)	0.0551 (6)	
O2	0.70120 (10)	1.19429 (14)	0.4881 (5)	0.0556 (6)	
H2	0.7320	1.2012	0.6036	0.083*	
Cl1	0.48481 (5)	0.70010 (7)	−0.1232 (2)	0.0753 (3)	
C1	0.89472 (15)	0.9967 (2)	0.6420 (7)	0.0592 (8)	
H1A	0.9204	1.0254	0.7930	0.089*	
H1B	0.9250	0.9996	0.4875	0.089*	
H1C	0.8809	0.9207	0.6755	0.089*	
C2	0.82884 (13)	1.0662 (2)	0.5953 (6)	0.0419 (6)	
C3	0.77663 (12)	1.03407 (19)	0.3905 (6)	0.0356 (6)	
C4	0.71379 (12)	1.09934 (19)	0.3463 (6)	0.0387 (6)	
C5	0.66330 (12)	1.06801 (19)	0.1590 (6)	0.0412 (6)	
H5	0.6227	1.1119	0.1321	0.049*	
C6	0.67293 (12)	0.97085 (19)	0.0103 (6)	0.0363 (6)	
C7	0.73511 (12)	0.9060 (2)	0.0441 (6)	0.0380 (6)	
H7	0.7426	0.8424	−0.0586	0.046*	
C8	0.78500 (13)	0.93830 (19)	0.2325 (6)	0.0385 (6)	
H8	0.8259	0.8947	0.2555	0.046*	
C9	0.62364 (13)	0.8441 (2)	−0.3163 (6)	0.0445 (7)	
H9A	0.6283	0.7797	−0.2001	0.053*	
H9B	0.6653	0.8470	−0.4320	0.053*	
C10	0.55550 (14)	0.8356 (2)	−0.4797 (6)	0.0506 (7)	
H10A	0.5519	0.9012	−0.5923	0.061*	
H10B	0.5591	0.7709	−0.5953	0.061*	
C11	0.48784 (15)	0.8259 (2)	−0.3180 (7)	0.0575 (8)	
H11A	0.4468	0.8274	−0.4368	0.069*	
H11B	0.4843	0.8899	−0.2002	0.069*	

### Atomic displacement parameters (Å^2^)

**Table:**

	*U*^11^	*U*^22^	*U*^33^	*U*^12^	*U*^13^	*U*^23^
O3	0.0445 (9)	0.0458 (10)	0.0476 (13)	0.0035 (8)	−0.0070 (9)	−0.0095 (10)
O1	0.0589 (12)	0.0490 (11)	0.0575 (14)	−0.0072 (9)	−0.0084 (11)	−0.0082 (11)
O2	0.0588 (12)	0.0425 (10)	0.0654 (16)	0.0090 (9)	−0.0098 (11)	−0.0183 (10)
Cl1	0.0772 (5)	0.0724 (5)	0.0764 (7)	−0.0267 (4)	−0.0077 (5)	0.0137 (6)
C1	0.0450 (16)	0.0682 (18)	0.064 (2)	0.0023 (12)	−0.0129 (17)	−0.005 (2)
C2	0.0448 (13)	0.0394 (14)	0.0414 (18)	−0.0095 (11)	−0.0003 (12)	0.0055 (14)
C3	0.0362 (12)	0.0317 (12)	0.0389 (16)	−0.0042 (10)	0.0019 (12)	0.0060 (12)
C4	0.0444 (13)	0.0289 (11)	0.0427 (18)	−0.0007 (11)	0.0035 (13)	−0.0005 (14)
C5	0.0403 (13)	0.0369 (13)	0.0463 (18)	0.0063 (10)	−0.0026 (13)	−0.0026 (13)
C6	0.0392 (13)	0.0358 (14)	0.0338 (15)	−0.0029 (11)	0.0041 (12)	0.0016 (11)
C7	0.0449 (14)	0.0301 (13)	0.0389 (18)	0.0003 (11)	0.0053 (12)	−0.0023 (12)
C8	0.0390 (13)	0.0312 (13)	0.0454 (18)	0.0021 (10)	0.0023 (12)	0.0052 (13)
C9	0.0467 (14)	0.0460 (14)	0.0408 (18)	−0.0031 (11)	0.0053 (13)	−0.0104 (14)
C10	0.0570 (16)	0.0529 (17)	0.0418 (18)	−0.0021 (13)	−0.0083 (14)	−0.0059 (15)
C11	0.0468 (15)	0.0565 (17)	0.069 (2)	−0.0035 (12)	−0.0072 (16)	0.0020 (16)

### Geometric parameters (Å, °)

**Table:**

O3—C6	1.361 (3)	C5—C6	1.393 (3)
O3—C9	1.452 (3)	C5—H5	0.9300
O1—C2	1.248 (3)	C6—C7	1.404 (3)
O2—C4	1.361 (3)	C7—C8	1.381 (4)
O2—H2	0.8200	C7—H7	0.9300
Cl1—C11	1.796 (3)	C8—H8	0.9300
C1—C2	1.500 (4)	C9—C10	1.515 (4)
C1—H1A	0.9600	C9—H9A	0.9700
C1—H1B	0.9600	C9—H9B	0.9700
C1—H1C	0.9600	C10—C11	1.504 (4)
C2—C3	1.467 (4)	C10—H10A	0.9700
C3—C8	1.402 (4)	C10—H10B	0.9700
C3—C4	1.424 (3)	C11—H11A	0.9700
C4—C5	1.382 (3)	C11—H11B	0.9700
			
C6—O3—C9	118.25 (18)	C8—C7—H7	120.5
C4—O2—H2	109.5	C6—C7—H7	120.5
C2—C1—H1A	109.5	C7—C8—C3	122.8 (2)
C2—C1—H1B	109.5	C7—C8—H8	118.6
H1A—C1—H1B	109.5	C3—C8—H8	118.6
C2—C1—H1C	109.5	O3—C9—C10	107.02 (19)
H1A—C1—H1C	109.5	O3—C9—H9A	110.3
H1B—C1—H1C	109.5	C10—C9—H9A	110.3
O1—C2—C3	120.6 (2)	O3—C9—H9B	110.3
O1—C2—C1	119.0 (3)	C10—C9—H9B	110.3
C3—C2—C1	120.4 (2)	H9A—C9—H9B	108.6
C8—C3—C4	116.8 (2)	C11—C10—C9	114.5 (2)
C8—C3—C2	122.5 (2)	C11—C10—H10A	108.6
C4—C3—C2	120.7 (2)	C9—C10—H10A	108.6
O2—C4—C5	117.7 (2)	C11—C10—H10B	108.6
O2—C4—C3	121.2 (2)	C9—C10—H10B	108.6
C5—C4—C3	121.1 (2)	H10A—C10—H10B	107.6
C4—C5—C6	120.3 (2)	C10—C11—Cl1	112.67 (19)
C4—C5—H5	119.9	C10—C11—H11A	109.1
C6—C5—H5	119.9	Cl1—C11—H11A	109.1
O3—C6—C5	115.1 (2)	C10—C11—H11B	109.1
O3—C6—C7	124.8 (2)	Cl1—C11—H11B	109.1
C5—C6—C7	120.1 (2)	H11A—C11—H11B	107.8
C8—C7—C6	118.9 (2)		

### Hydrogen-bond geometry (Å, °)

**Table:**

*D*—H···*A*	*D*—H	H···*A*	*D*···*A*	*D*—H···*A*
O2—H2···O1	0.82	1.85	2.570 (3)	146
